# Editorial: Catechol and Polyphenol Chemistry for Smart Polymers

**DOI:** 10.3389/fchem.2019.00883

**Published:** 2019-12-20

**Authors:** Bruce P. Lee, Henrik Birkedal, Haeshin Lee

**Affiliations:** ^1^Department of Biomedical Engineering, Michigan Technological University, Houghton, MI, United States; ^2^Department of Chemistry, Aarhus University, Aarhus, Denmark; ^3^Department of Chemistry, Korea Advanced Institute of Science and Technology, Daejeon, South Korea

**Keywords:** catechol, polymer, smart materials, polyphenol, interface

This special issue of *Frontiers in Chemistry* features papers that utilize phenolic compounds such as catechols for designing functional coatings and materials. Recently, scientists worldwide have incorporated catechols and various phenolic compounds in designing advanced and multifunctional materials with unique properties (e.g., self-healing, underwater adhesion, stimuli responsive properties, etc.) (Forooshani and Lee, 2017; Andersen et al., 2019). These materials have potential in a wide range of applications in the biomedical, energy, industrial, and possibly other fields. The reasons for the wide adoption of phenolic compounds in material design is due to the unique ability of these compounds to participate in both irreversible and reversible interactions. This diversity in chemistry enable these phenolic compounds to function as adhesive primer to wide ranges of surfaces. Most importantly, by simply functionalizing polymeric materials with phenolic compounds, these initially inert materials are imparted with the unique properties (e.g., adhesive property, redox active, etc.) of these phenolic compounds.

Jalaber et al. employed atmospheric aerosol assisted pulsed plasma process to directly polymerize a catechol-modified monomer, dopamine acrylamide, and directly deposit catechol-containing thin films on to a surface. This process occurred in a dry and catalyst-free condition, and overcame limitations associated with polymerization in a solution that often resulted in poor wetting and the formation of inhomogeneous coatings. The plasma discharge rate and timing were used to tune the morphology, deposition rate and catechol content for creating homogeneous catechol-bearing polymer thin films.

The formation of polydopamine coatings involves the autoxidation and crosslinking of dopamine to form an adhesive primer for additional surface functionalization (Ryu et al., 2018). Alfieri et al. reported the effect of aliphaticdiamine, hexamethylenediamine (HMDA), on the polymerization process of dopamine to form a polydopamine coating. HMDA promoted intermolecular crosslinking between the oxidized form of catechol and subsequently promoted film deposition. This article further confirmed the use of resorcinol as an inhibitor to polydopamine formation.

Forooshani et al. utilized polydopamine to design antimicrobial coatings that releases the disinfectant, hydrogen peroxide (H_2_O_2_) when the coating was hydrated in an aqueous solution. H_2_O_2_ is generated as a byproduct during catechol autoxidation (Meng et al., 2015). Polydopamine was coated onto inert polypropylene mesh, which is often used in soft tissue reconstruction surgery. A two-step coating approach was used to optimize the coating for H_2_O_2_ generation and the generated H_2_O_2_ was effective against both gram positive and negative bacteria.

Tyo et al. investigated the effect of H_2_O_2_ generated from polydopamine coating on *Psychrobacter cryohalolentis*, the most common bacteria found on the surface of humpback whale skin. The generated H_2_O_2_ was found to be biostatic against *P. cryohalolentis* and reduced its attachment. Polydopamine coating can potentially be used to impart antimicrobial properties to satellite telemetry tags used for tracking the migration patterns of large cetaceans, which is a critical first step in the conservation of these animals.

Kim et al. provided a mini-review that discussed the pro- and antioxidant properties of catechol containing chitosan films. This review describe the methods of fabrication and characterization of the film. Conditions that promoted either the antioxidant or pro-oxidant behavior of the redox active catechol film were also reviewed, which can potentially be useful for designing functional materials aimed at radical scavenging activities or antimicrobial functions.

Yang and Lee utilize the adhesive property of catechol to create a lubricious, antifouling hydrogel coating to prevent the binding of bacteria to urethral catheter. Catechol was chemically linked to three types of biopolymers (chitosan, hyaluronic acid, and human serum albumin) and coated onto various elastomers commonly used to fabricate urethral catheters. The coating significantly lowered the friction coefficient of the elastomers. Additionally, incorporation of silver nanoparticles into the coating enhanced the antimicrobial property of the coating.

## Author Contributions

All authors listed have made a substantial, direct and intellectual contribution to the work, and approved it for publication.

### Conflict of Interest

The authors declare that the research was conducted in the absence of any commercial or financial relationships that could be construed as a potential conflict of interest.
